# Adaptive Rehabilitation Bots in Serious Games †

**DOI:** 10.3390/s20247037

**Published:** 2020-12-09

**Authors:** Imad Afyouni, Abdullah Murad, Anas Einea

**Affiliations:** 1 Department of Computer Science, University of Sharjah, Sharjah P.O. Box 26666, UAE; 2 College of Computer and Information Systems, Umm Al-Qura University, Makkah 21421, Saudi Arabia; aamurad@uqu.edu.sa; 3 PROS Corporate, Houston, TX 77098 USA; anasainea@gmail.com

**Keywords:** serious games, motion capture, TeleRehabilitation, user adaptation, intelligent alerting, automatic correction

## Abstract

In recent years, we have witnessed a growing adoption of serious games in telerehabilitation by taking advantage of advanced multimedia technologies such as motion capture and virtual reality devices. Current serious game solutions for telerehabilitation suffer form lack of personalization and adaptiveness to patients’ needs and performance. This paper introduces “RehaBot”, a framework for adaptive generation of personalized serious games in the context of remote rehabilitation, using 3D motion tracking and virtual reality environments. A personalized and versatile gaming platform with embedded virtual assistants, called “Rehab bots”, is created. Utilizing these rehab bots, all workout session scenes will include a guide with various sets of motions to direct patients towards performing the prescribed exercises correctly. Furthermore, the rehab bots employ a robust technique to adjust the workout difficulty level in real-time to match the patients’ performance. This technique correlates and matches the patterns of the precalculated motions with patients’ motions to produce a highly engaging gamified workout experience. Moreover, multimodal insights are passed to the users pointing out the joints that did not perform as anticipated along with suggestions to improve the current performance. A clinical study was conducted on patients dealing with chronic neck pain to prove the usability and effectiveness of our adjunctive online physiotherapy solution. Ten participants used the serious gaming platform, while four participants performed the traditional procedure with an active program for neck pain relief, for two weeks (10 min, 10 sessions/2 weeks). Feasibility and user experience measures were collected, and the results of experiments show that patients found our game-based adaptive solution engaging and effective, and most of them could achieve high accuracy in performing the personalized prescribed therapies.

## 1. Introduction

As of late, the challenge of designing a highly engaging serious game has been a subject of interest to various fields including training, simulation, healthcare, and education, among others. Specifically, the direction towards creating a home-based individualized training plan for patients diagnosed with mental and/or physical disorders has attracted more interest due to the recent advances of technologies and techniques related to gamified telerehabilitation. Traditionally, physical therapy solutions present several challenges. First, the patient-to-therapist ratio for physical rehabilitation is generally unable to comply with the ideal guidelines for staffing assumptions [1]. This usually results in appointment delays and reduced treatment time. Second, patients are, in most cases, required to perform additional exercises at home, allow timely recovery, and reduce the recurrent expensive visits to hospitals or rehabilitation centers [2]. Third, traditional therapy methods require the supervision and monitoring of experienced professionals in order to assess patients’ performance and improvement over time. However, home-based exercises are considered as non-attractive, and are kept unmonitored without advanced analytics that allow measuring the patient’s key performance indicators [3].

**Figure 1:**
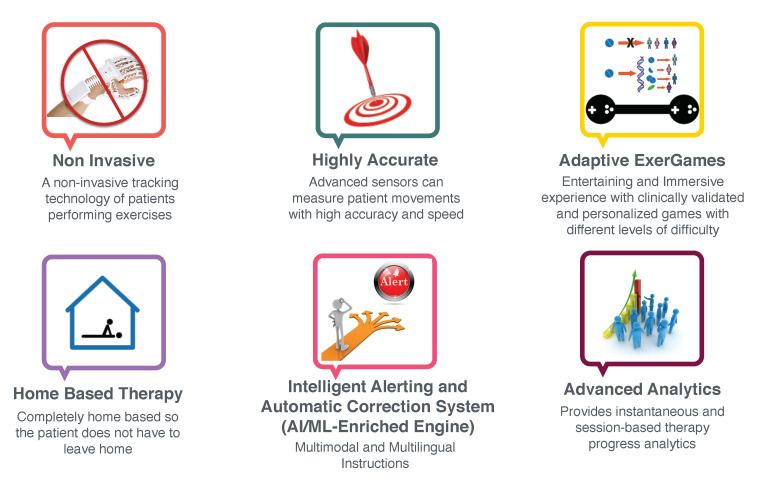
Required features for building adaptive serious games.

We believe there are some unique features that can overcome the mentioned challenges, which are illustrated in Figure 1. Advanced 3D motion capture devices can lay the groundwork for developing noninvasive and home-based physiotherapy solutions, while maintaining highly accurate monitoring of patient’s performance in real-time. On the other hand, a successful telerehabilitation platform should provide instantaneous and session-based therapy progress analytics. Moreover, using the power of AI and intelligent algorithms, a personalized set of exercises should be developed within an adaptive serious game framework, which we refer to as “adaptive exergames”, so that patients can have an entertaining and immersive experience with clinically validated and personalized games that adapt to different levels of difficulty.

Consequently, we introduce the “RehaBot” system to overcome the challenges presented by the traditional therapy solutions, and it differs from existing work in many ways: (i) RehaBot is comprehensive in that it was not designed for rehabilitating specific body parts, but rather it accepts various therapies to cover the whole body; (ii) in addition, RehaBot embeds virtual assistants to direct patients to perform the exercises correctly through 3D illustrations, while in case of failure, it indicates the joints/gestures that need improvement; (iii) furthermore, RehaBot is intelligent in assessing patients’ range of motions of various body parts and then adjust the game’s level of difficulty in real-time tailored to the patients’ abilities, so that they do not lose interest of the game due to unrealistic difficulty levels. A variety of upper limb and lower limb disabilities can be covered in this platform due to the use of motion caption sensors, such as the MS Kinect 2, which covers the body joints starting from head, neck, spine and upper body joints, to knees, foots, and ankles. However, in our evaluation we have focused on upper limb disabilities, including cervical spine diseases, and lower back pain, as they are very common among all ages and the access to patients dealing with such disorders is much easier than other types of disability.

This paper extends our previous work introduced in [4,5] by demonstrating all algorithmic details behind our posture matching, smoothing, and adaptation techniques in “RehaBot”, and by conducting a clinical study to investigate the impact of this adaptive game-based physiotherapy solution on patients dealing with chronic neck pain as a case study. We believe, to the best of our knowledge, that there is no similar work in literature that integrates adaptive virtual assistants in real-time within serious games for rehabilitation. The main contributions in this paper are summarized as follows.

- RehaBot embeds real-time pattern and gesture recognition and different adaptation techniques to tailor to patients’ preferences and needs.
- The game engine incorporates three levels of adaptation: (1) an embedded virtual assistance, a rehab bot, that shows the users how to perform the exercises correctly; (2) a module of intelligent notifications that reads and represents the patient postures/movements in 3D, then if needed, adjusts the game to the appropriate level that ensures a better outcome; and (3) a dynamic correction module that takes into consideration both the game level of difficulty and the virtual assistant readings to produce a tailored set of exercises that are more appropriate to the patients’ abilities; thus, reducing the gaps between the current range of motions patients are able to perform and the ideal suggested ones.
- Advanced real-time and session-based analytics are also generated to allow for automatic learning of patient behavior over the different sessions.
- A fully-fledged system was developed with both therapist and patient dashboards. 3D motion capture and VR daemons will be running on patient’s front-end with a possibility to adapt game configuration and controls in a seamless way based on performance.

It should be noted that while the system implements its functionality using the MS Kinect 2 and HTC VR headsets, these devices do not constitute an essential factor for the platform and can be replaced by other alternatives (such as Intel RealSense and Oculus Rift) if needed. Indeed, while motion sensors provide data about joint positions and vector orientations, VR devices report the position and orientation blueprints of the headset. Different case studies including upper or lower limb disorders can be considered to evaluate the performance of the proposed system, as our employed motion tracking device (i.e., Kinect) can track 25 joints covering the full body. The presented clinical trials focus on patients with chronic neck pain with the aim of having a homogeneous group of patients. However, this study can be easily covering other common cases in upper limb disorders, such as lower back pain and scoliosis.

The remainder of this paper is organized as follows. Section 2 discusses the related work in serious games for telerehabilitation. Section 3 first presents the smoothing filters employed in motion tracking, then introduces the different adaption techniques towards the generation of personalized and adaptive serious games. Section 4 presents the system development and usage, and describes the salient components of the system architecture. Section 5 presents the implementation details, while Section 6 describes the methodology of the clinical study and highlights the evaluation process towards proving the effectiveness and usability of our system. Finally, Section 9 concludes this paper with a discussion on future work.

## 2. Related Work

Recent studies explored the possibility of utilizing patient-centered telerehabilitation as an adjunctive therapy to overcome the challenges in conventional therapy solutions [6,7]. Within this context, serious games appeared to be a promising candidate in bridging that gap, by providing an engaging environment to perform the required exercises, while using 3D monitoring devices that allow tracking of patients’ gestures and performance in real-time [2,8]. Remote rehabilitation of patients dealing with musculoskeletal disorders through serious games allows for close guidance and encourage patients towards performing online low-cost physiotherapy exercises that can help them live independently at home [9]. In its attempt to improve the physical therapy outcomes, the human–computer interaction (HCI) community has proposed using several new interactive tangible and intangible devices together with serious games [10,11]. The authors of [12] show that serious games are effective for the physical therapy of stroke patients. This is also resulting from the fact that the more time is spent in rehabilitation, the greater the extent of recovery. The authors of [13,14] designed a game-based system for detecting, tracking, and visualizing joint therapy data. Afyouni et al. [15] developed a game to be played with a Leap Motion controller for rehabilitation of the hand movement of upper extremity for the stroke patient. Jonsdottir et al. [16] designed a game framework for arm rehabilitation to motivate patients dealing with multiple sclerosis. The authors of [7,17] presented reviews on serious games for motivating body exercises in case of stroke and physical rehabilitation. Another approach for cognitive rehabilitation using a serious game platform was proposed in [18]. More specific serious games for physical rehabilitation of patients with chronic musculoskeletal back and neck pain were proposed in [19], by using the motion capture (MoCap) system and virtual reality.

Several challenges need to be addressed while designing serious game solutions in physical rehabilitation, as discussed in [20]. These challenges need to be considered for developing successful serious games in general and were adopted as guidelines in our framework design. They are categorized within different phases as follows.

1. The pre-design phase consists in analyzing the needs, audience, best technologies to be used, goals and outcomes, and assessment tools for measuring the performance.
2. The design phase covers the development of all necessary components in the serious game, while considering factors such as game interactivity, enjoyment, clarity, and user experience. We believe that adaptiveness of the serious game to patients’ needs and performance should also be taken into account at this stage.
3. Finally, the evaluation phase, which consists in measuring the outcomes achievement and the user experience. With respect to physical rehabilitation, outcomes are related to skills acquired and range of motion improvement. Outcomes are considered as achieved when the patient performs the prescribed training correctly and with no side effect on his/her overall medical conditions. Measuring the user experience should also cover the satisfaction level and usability of the proposed serious game.

Additional challenges related to the development of evaluation guidelines were also discussed in [21]. The evaluation guidelines consist in defining several evaluation metrics, the process towards conducting the clinical trials, the analysis of results and feedbacks, and the improvement of the game design.

### 2.1. 3D Motion Capture and Virtual Reality

Several devices can be used to capture 3D motions and identify the physiological features from a rehabilitation session, such as Intel Realsense, Microsoft Kinect, StereoLabs Zed https://www.stereolabs.com/zed/, and Orbbec https://orbbec3d.com. When integrated with algorithms of skeleton pose tracking and point cloud, these sensors can assess various types of physical functions and anatomy [22,23]. Others have used Vicon cameras to recognize gestures with aids of markers and computer vision technology https://www.vicon.com/products/camera-systems. However, unlike Microsoft Kinects [24,25], Vicon cameras are not widely adopted in healthcare studies due to the high price and level of complexity to set them up at patients’ homes. Kinect 2 is well known for its ability to track 25 joints simultaneously allowing it to capture the full human body gestures. However, Kinect 2 sometimes falls short when it comes to (1) capturing subtle hand motions with high accuracy, (2) recognizing short-range motions, (3) functioning in bright sun light, and (4) immunity to interference caused by other nearby sensors [26]. A recent work [27] demonstrates a superiority in pose tracking performance of the Azure Kinect over the Kinect 2, in which the Azure Kinect more accurately measured the spatial gate parameters. Improving joint position estimation of Kinect using various types of filters can greatly enhance the rehabilitation experience [28].

Virtual reality-based exergames for rehabilitation were proposed in several studies. The aim behind these studies is to identify the advantages and barriers perceived by experts to using an immersive Exergame as an adjunctive treatment to conventional therapy. VR-based serious games are designed using VR headsets, which are head-mounted displays (HMDs) integrating inertial measurement units (IMUs). The two branded VR headsets that are mostly used are Oculus Rift (in its three versions) and HTC Vive, in addition to other low-immersion solutions such as cardboards or Gear VR. The use of 3D motion tracking sensors and virtual reality headsets in rehabilitation has been investigated in [29,30,31], among others. A comprehensive review of developed serious games for learning and training purposes in immersive VR-environments was presented in [20]. The proposed studies discuss different types of exercises by which the patient can train or rehabilitate several aspects such as musculoskeletal or cognitive disorders. These studies commonly suggest that using virtual reality exergaming technology as an adjunct treatment to traditional therapy is engaging and safe for post-stroke rehabilitation and can be beneficial to upper extremity functional recovery. A recent work on designing rehabilitation exercises for chronic neck pain using serious games and virtual reality was also proposed in [19]. However, a few limitations have been highlighted on the use of immersive VR-based serious games. For instance, virtual reality devices might result in some sort of motion sickness, referred to as *‘1cybersickness”*, mostly resulting from conflicting signals about the body’s orientation and motion [32]. Checa and Bustillo [20] suggest that this VR sickness syndrome might be diluted by considering new strategies for user interaction and the development of rich storytelling in the VR-based serious games. Maier et al. [33] also suggest to build neuro-scientifically grounded protocols for more effective VR-based interventions. Another important challenge is related to the evaluation of such VR-based serious games, and especially related to the number of subjects that are recruited for testing purposes [20]. Having a sufficient number of patients will increase the statistical significance of the study, but usually in physical rehabilitation, the size of the target group is very limited. Finally, existing works focus on better human interaction with mounted devices and generic games for all patients. Therefore, they lack the integration of patient-centered techniques that learn from user’s current and past behavior and performance, thus adapting the gaming environment to their needs.

### 2.2. Adaptation in Serious Games

Games can be adapted in various ways to create a user experience that is both personalized and customized. In order to create an adaptive game, a dynamic process of adjustment of the game components, such as user interfaces and strategies, game mechanics, and difficulty levels, has to be embedded [31,34]. Some games assess the user’s performance at the beginning and adjust the game upfront, while others continuously adjust the game during sessions [35]. There are two types of game difficulty level adaptation: static or dynamic. A static difficulty level implies that the parameters of the game are adjusted based on a predefined set of formula that increase/decrease the difficulty levels of the game as cited in [36]. A game-based exercising program in health sport, presented in [36], is an example of a game’s static difficulty level, in which the workout settings and the individualized needs of the players are preset. By contrast, the dynamic difficulty levels require close monitoring of users’ performance along with a wide range of parameters that can be dynamically adjusted [37]. Thus, the more parameters that can be adjusted, the better game adaptation can be achieved. Furthermore, the adaptation techniques that are fully dynamic make the performance of home-based therapy sessions much easier compared to the static ones [38].

Nonetheless, there is one drawback associated with adopting a fully dynamic gaming environment in that it may not leave enough room for therapists to test their therapeutic plan in a controlled environment aside from other factors. Thus, the semi-dynamic approach enables therapists to adjust non-related game parameters, while leaving the adjustment of the game-related parameters to the system based on the performance of the patients. An example of a game that was designed using a semi-dynamic approach is the game of catching falling fruits [39], in which the system controls the falling fruits’ number, size, weight, fall frequency, and the number of baskets needed to collect them. On the other hand, the non-game-related parameters controlled by the therapist are the exercise type, session period, number of repetitions, and movement constraints. Another example is the serious game environment presented in [40], in which the therapist provides real-time guidance allowing for live interactions with the patients and thus better performance. A third example is the adaptive hand therapy game created in [41], in which the therapist’s prescription is translated into a 3D path which the patient has to follow to complete the therapy exercise. A fourth example is an algorithm used in the education field to produce personalized learning lessons using a Kinect-based system [42]. A fifth example presented in [9] demonstrates a system that uses the user’s interactions, previous performances, and preferences to recommend new game-based exercises.

Although the presented approaches consider adaptation techniques from different perspectives, none of them focused on automatically generating adaptive game configurations and embedding adaptive virtual characters from therapeutic instructions, which is a major concern in our work. Moreover, full body posture adaptive design and matching, as well as self-adaptivity of our rehab bots based on patient performance, were not previously discussed. In this work, we have also adopted a semi-automatic adaptation strategy whereby an expert can author a set of recorded postures along with a few non-game-related parameters (e.g., number of repetitions or exercise duration), while the system takes that input and thoroughly generate new game configurations specifically designed to fit that particular patient.

### 2.3. Existing 3D Motion Capture Systems from Market Perspectives

**Figure 2:**
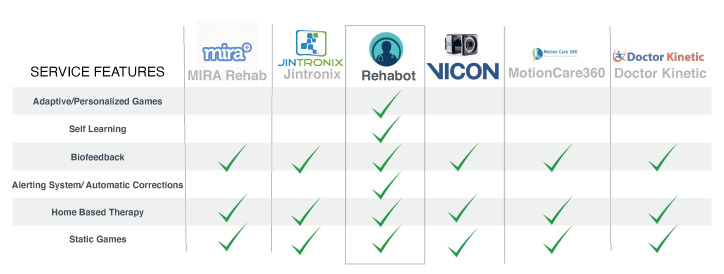
Evaluation of the closest related works in literature.

A market study demonstrates an increasing interest in remote physiotherapy solutions. A number of companies have implemented and presented various platforms for 3D motion capture with applications in different domains from sports, to entertainment, virtual reality, and physical rehabilitation. Figure 2 illustrates a list of relevant systems that are assessed with respect to a set of criteria we believe are important for an effective adaptive serious game platforms. Those systems, including Mira from Mira Rehab www.mirarehab.com/, JRS from Jintronix for Kinect-based rehabilitation www.jintronix.com, Vicon from the Oxford Instruments Group www.vicon.com/, MotionCare360 www.mobihealthnews.com/tag/motioncare360, and Doctor Kinetic doctorkinetic.com, are among remote physiotherapy solutions that employ relatively tangible and intangible sensors for tracking body joints and measuring therapy compliance. However, to the best of our knowledge, none of these systems embed dynamic techniques for designing personalized and self-adaptive serious games, such as the ones proposed in our Rehabot system.

## 3. Adaptive Game Generation

The progression of creating adaptive serious games for therapeutic purposes, also called exergames, has been accelerated after the advance monitoring devices surfaced. Exergames allow patients to take part in a customized, enjoyable, and immersive sessions, while conducting clinically validated training exercises. Adaptive exergames assess patients needs and capabilities to automatically generate personalized sets of training exercises. The challenges in developing adaptive serious games include (1) transforming recommended postures by an expert into a series of virtual assistants exhibiting those postures in the right place, time, and number of occurrences within the game environment; (2) implementing an accurate real-time posture mechanism that allows guiding the patient towards performing the right exercises and detecting the imperfect gestures within and over sessions; and (3) redefining the rehab bots’ behavior by adapting the game level of difficulty to suits patients needs and performance.

Rehabot embeds adaptation techniques at different levels to tailor to the patient’ profile. The automatic adaptation requires integrating *“virtual coaching”* into the gaming environment to ensure that patients are performing the exercises correctly. Such an integration is associated with three levels of adaptation techniques as mentioned above and described in Section 3.2, Section 3.3 and Section 3.4, respectively.

### 3.1. Smoothing Filters for the Kinect Tracking System

3D motion capture sensors and virtual reality head-mounted displays (HMDs), namely, the Microsoft Kinect 2 and HTC Vive, are used in this platform, thus leveraging real-time monitoring of patients’ gestures and activities. Nonetheless, the tracking system of body postures in Kinect presents noisy data and is sometimes unstable, especially when multiple joints appear in front of each other [28,43,44]. Consequently, the first objective in this work was to employ smoothing and noise reduction filters, so that joint position and orientation remain stable over time.

In this phase, the accuracy of the Kinect data has undergone several filtering stages in order to achieve the best performance. It is worth mentioning that the Kinect device might show inconsistent data, especially when two body joints appear on top of each other. In this case, the hidden joints’ positions might be predicted in a wrong way. The filtering process aimed at smoothing these inconsistencies by removing unreliable data streams, or by applying extrapolation between previous positions of the same joint in order to predict the right next position. Different filtering algorithms for improving joint position estimation of Kinect have been proposed, such as, low-pass filtering, double exponential filtering, and Kalman filter [28,44]. Due to the dynamic nature of our tracking system, we propose to a two-stage filtering process: the first stage applies double exponential filtering using Holt’s method to skeleton data as time-series data [45]. Then, in a second phase, the smoothing process uses a statistical analysis to create a moving average of joints data, thus reducing outliers from the generated frames. The double exponential filtering proves to be working well with joint movements that do not interfere with each other. However, the double exponential filtering does not consider the nature of the joint, its location within the body, and the occurring movement in real-time. Consequently, a second filtering stage was added, which evaluates the distances between each joint and its parent joint, and keeps track of the history of joint positions and orientations in order to predict the best new values considering the value input form phase 1. We believe this double-stage filtering can naturally understand human motion, thus providing us with smooth body tracking in highly dynamic situations when compared to other filtering approaches.

This process shows a better stability and reasonable results even in highly dynamic situations with multiple joint interference. Algorithm 1 describes our developed filtering process. The algorithm takes the Kinect frames, joints names and their distances to parents, and joint historical positions and orientations. Typically, we consider the last frames in a given temporal range for historical positions and orientations, so that we can detect the pattern and movement evolution for each joint. This algorithm uses the double exponential filter in its first phase, then performs extra calculations and validation to produce the updated frame as an output in the second phase. The detailed steps are highlighted as follows.

- In the first phase, each frame should undergo a double exponential smoothing to ensure the series of data for joint positions exhibit some form of trend without outliers (line 3). This smoothing filter takes the historical datasets for joint positions and orientations up to a certain instant *t* in order to estimate the best value for the joint position at the instant t+m. The filtering parameters include the ‘data smoothing factor’ that defines how much the predicted value should remain close to raw data, the ‘trend smoothing factor’ which takes the historical evolution of the data series over time, the ‘jitter radius’ for jitter reduction, and the ‘maximum deviation radius’ which depicts the distance that filtered positions are allowed to deviate from raw data. The output of this stage is an updated frame with smoothed series of values estimated according to the mentioned parameters.
- The second phase consists in evaluating the estimated values obtained from the double exponential filtering, by considering two main factors: the distance between each joint and its parent, and the maximum angular velocity for each joint. The set of distances between joints is determined statically, whereas the maximum rotation is calculated for each joint based on its motion nature and the historical records. Lines 5–10 highlight this process for each joint. First, the algorithm determines the joint parent and calculates the distance difference between the two joints in the current frame data; then, it evaluates the mismatch with the static distance between the joint and its parent $\epsilon_{j_{k}}$. Similarly, the angle rotation is retrieved from the current frame, and compared to the maximum angular rotation estimated $\delta_{j_{k}}$. The algorithm then estimates the percentage of error based on these two parameters, and decides based on a given threshold whether the current predicted value for each joint can be trusted.

The output of this algorithm is the new set of frames with updated predicted series of values. Only values of joint positions and orientations that are not trusted, are marked with a low confidence level, which is taken into account in posture matching as described in Section 3.2.
**Algorithm 1:** Kinect Data Smoothing Algorithm.
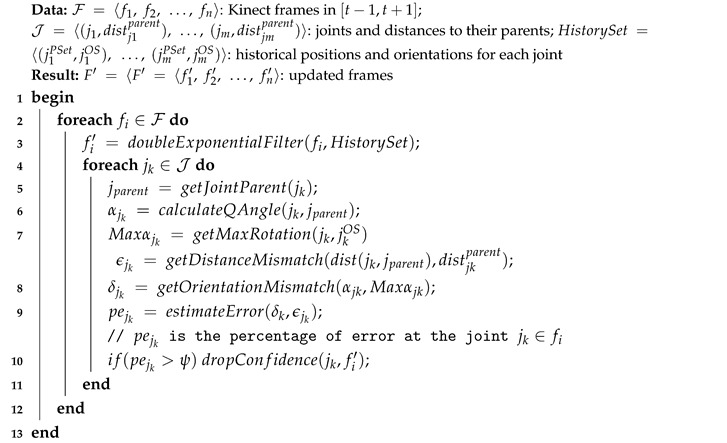


### 3.2. Posture Matching

To start the exergame, a therapist inputs a set of correct postures for the corresponding patient to practice. The system then identifies a set of joints that are used with each inputted posture and starts monitoring them. In order to identify the joints, the system compares the correct posture with the human body in an idle position, and identified joints are given more weights as opposed to other unidentified body joints. mathcalW indicates the set of weights that is assigned to the identified body joints to be monitored. The significance of each joint with respect to a specific posture is represented by its assigned weight.

A posture matching technique is developed to calculate the closeness indicator with the ideal posture displayed by the virtual character, so that patients’ joints are compared against their ideal counterparts following the specifications prescribed by the therapist. The posture matching technique is described in Algorithm 2. The algorithm takes, as inputs, the values of F, BP, and W, which depict the patient current frame, the correct bot posture, and the set of weights that indicate the significance of the identified joints, respectively. The algorithm returns, as an output, a series of 3D quaternion indicators calculating the differences for all body joints together with the error percentage for each joint compared to the correct gesture. Moreover, the system returns the overall percentage of matching. More details are presented next.
**Algorithm 2:** Posture Matching Algorithm
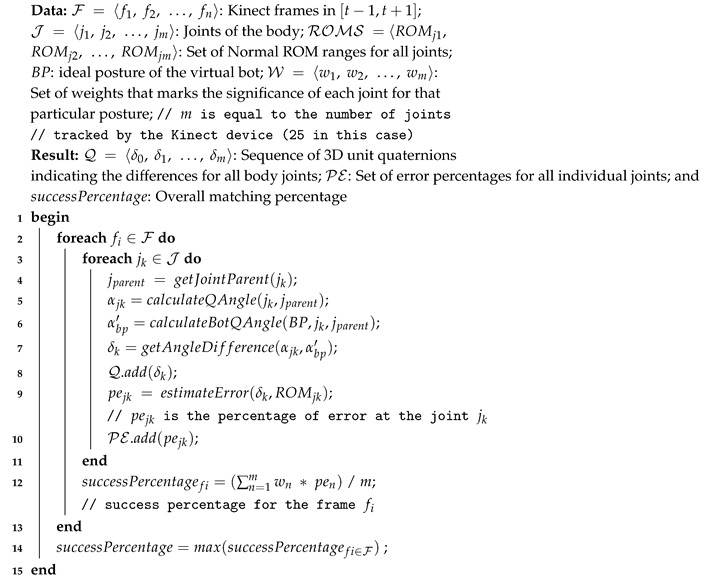

**Figure 3:**
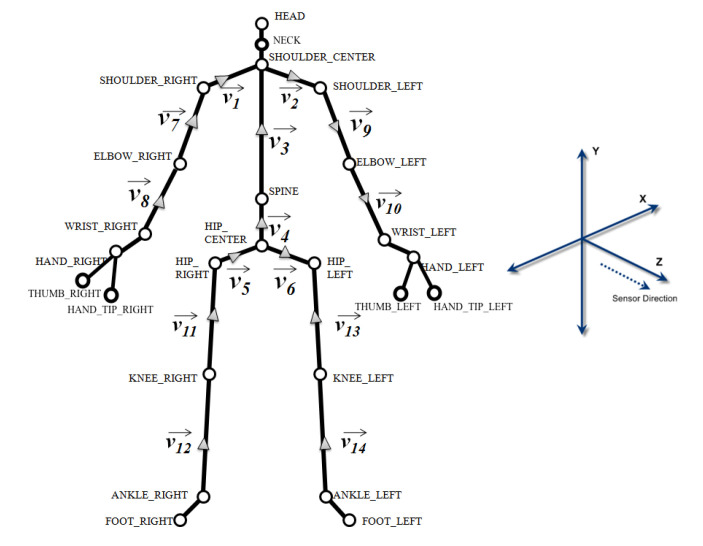
Body joints and vectors as detected by the Kinect sensor.

**Figure 4:**
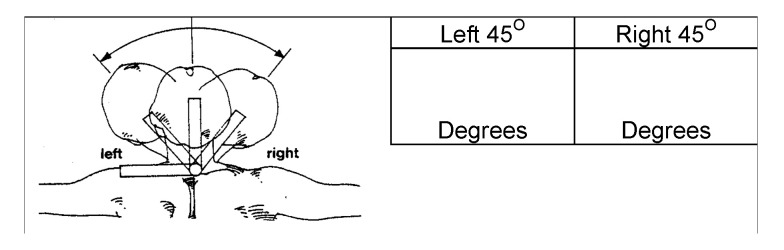
Neck lateral bending range of motion interval.

- The matching process launches when the patient reaches the next virtual bot posture range. The matching process calculates all the frames that are recorded within the [t−1,t+1] time frame, where *t* represents the appearance timestamp of the virtual bot (Line 2).
- The system returns all joint positions and their respective directions for each frame in the form of 3d vectors relative to each bone. As demonstrated in Figure 3, MS Kinect 2 tracks continuously 25 body joints. For each bone, the system computes the 3d axis-angle, such as the quaternions unit $\alpha_{jk}$ that links the parent bone, such as leg with hip, with the bone (Lines 3–5). The system also calculates the equivalent unit quaternion of correct posture as suggested by the virtual bot, such as $\alpha_{bp}^{\prime }$ and then determines the 3d angel difference $\delta_{k}$ of that specific joint (Lines 6–7).
- The system also computes for each frame the overall matching percentage between all bones from the frame of the patient and their corresponding ones from the registered posture of the virtual assistant. The matching percentage is calculated by dividing the 3d ratio of rotational deviation over the interval of range of motion. For instance, Figure 4 shows the estimated neck lateral bending ROM is between ($-45^{\circ }$ (left), $45^{\circ }$ (right)). Thus, the estimated percentage of errors is 33% resulting from the 15% deviation of the neck lateral bending towards the right. In this manner, for each bone, the system starts the matching process at the local scale, then calculates the nearest indicator for every vector, after that produces an abstracted matching indicator of the whole body posture relative to the virtual bot (Lines 9–10).
- Finally, for each frame, the system calculates the final score and compares it with the score of the correct posture that comes from the virtual assistant (Line 12). Then, the best frame within range is selected and compared to the threshold value (i.e., 85% success rate) (Line 14). If the score is greater than the threshold value, it is considered a success; otherwise, the system will suggest guiding instructions to help the patients improve their performance.

### 3.3. Intelligent Alerting

In order to provide the user with real-time guidance, Rehabot takes advantage of the closeness indicator computed previously and recommends multimodal instructions towards improving the patient performance while exercising. Computing such alerting instructions is done by analyzing the positions and orientations of the whole body joints. As a result, the system can determine the deviation from the ideal posture at a high level, but also can estimate detailed joint deviations, so that the patient can understand exactly if something goes wrong. Estimating the deviation in direction and 3D angular rotation of each joint with respect to the virtual bot in real-time is challenging. An example instruction that is expected would be “move your left wrist up/right by 45 degrees”. Algorithm 3 highlights the different steps towards generating real-time guidance and feedback on current performance.
**Algorithm 3:** Intelligent Alerting Algorithm.
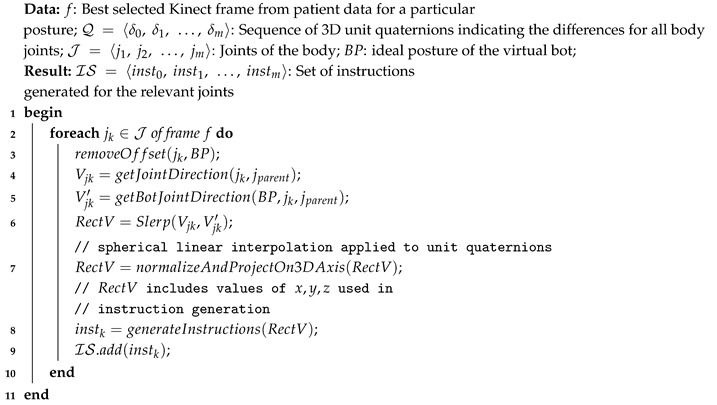

- We evaluate the posture matching between the virtual bot and the patient posture in real-time. If the closeness indicator representing the overall posture deviation is less than a specified threshold (e.g., 85%), then the system starts a detailed investigation and reports the least performing gestures.
- For each joint in the detected posture frame, the system determines the deviated angle on the 3D axis between the parent bone of the patient and the corresponding one from the virtual bot, then the 3D angle is adjusted by removing the generated offset from the rotation of the user bone, thus realigning joint directions between the user and the bot, and removing any initial offset of the parent bone (Lines 2–3).
- Lines 4–6 explain the process for computing the direction of movement vectors for both the patient and the virtual assistant. The system then spherically interpolates the rotation between both vectors, and calculates the straightest and shortest paths between its quaternion end points $V_{jk}$ and $V_{jk}^{\prime }$ through the *Slerp* function.

This quaternion path is usually referred to as the “rectification” vector RectV. Normalization is then applied to RectV, in order to determine the axes used in the recitifcation process (i.e., estimating in which direction the user has to move his/her bone). For example, a positive value on x axis suggests moving the corresponding bone to the right, while a negative value would require moving it to the left (line 7).

There, we apply the same technique on up/down directions, so that we can generate relevant instructions in order to improve the patient’s gestures for each exercise (Lines 8–9). Generally, to avoid an overwhelming information load, instructions are focused on the three least performing gestures when evaluating the posture in real-time. Displayed instructions include the joint description, desired direction (e.g., up|right), and angle in degrees.

### 3.4. Automatic Correction

Different groups of gestures are evaluated separately over several occurrences of gestures of the same type, and recommendations are generated by the system in real-time to improve the patient performance. However, in case the overall performance has been improving after several attempts, the virtual assistant acts as an adaptive character that can adjust itself to match to patient capabilities. This can be done in a reverse way to what has been described in Algorithm 3. Indeed, Rehabot takes advantage of the sequence of 3D angle differences for all body joints Q over the last attempts, as well as the calculated rectification vectors. The purpose is to minimize the gaps calculated in rectification vectors of the relevant joints (i.e., those with high precalculated weights) by reducing the rotational difference in the monitored joints by a threshold ε. As a result, an updated posture with a lesser difficulty level is generated and shown by the virtual assistant in the next attempts.

This automatic verification of patient performance is performed after a minimum of three attempts for each group of gestures. If the patient is unable to perform as expected after those attempts, the system decides to automatically decrease the difficulty level for the assessed posture or group of gestures to maintain a convenient level of difficulty. On the other hand, and upon the successful completion of a group of gestures, an upgraded level of difficulty is generated by creating a complementary set of gestures until reaching the best performance for a given exercise as prescribed by the therapist. Therefore, virtual bots are adapted by generating updated postures that are slightly superior or inferior with respect to the previous postures based on patient performance. This helps in keeping the gaming environment enjoyable, while pushing the patient towards a better performance.

## 4. System Development and Usage

**Figure 5:**
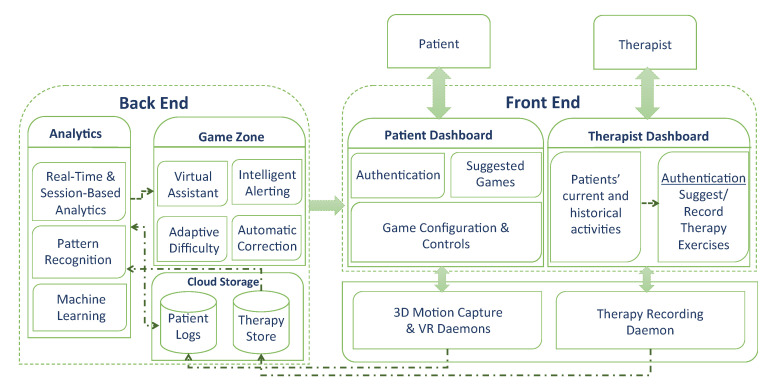
System architecture.

This section demonstrates the system architecture overview and discusses how its two types of users—therapists and patients—can interact with it. The aim of this platform is to produce a serious game for physical rehabilitation that is proactive, adaptive and personalized. Figure 5 demonstrates the three essential components of the platform. These are (1) the frontend component, which includes dashboards for the patients and therapists as well as the adaptive game; (2) the processes component, which includes all the processes that run in the background and collect data from the therapists and patients; and (3) the backend components that comprise the intelligence of the game’s engine, the three methods of analytics, and the storage residing at the cloud.

**Figure 6:**
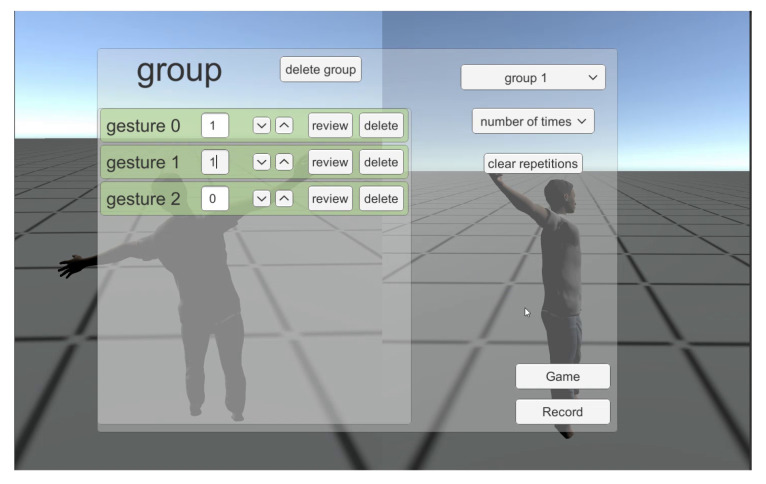
Dashboard for therapists to input groups of gestures.

As shown in Figure 5, two dashboards are created to attend to the therapists’ and patients’ needs. The therapists use the dashboard to record gestures and input prescriptions. Then, the therapists assign the appropriate exercises to the patients based on their capabilities and needs (see Figure 6). In order to record gestures, therapists need to use a 3D motion capturing sensor such as Kinect XBOX. Moreover, therapists use the dashboard to manage and monitor patients’ progress and performance over time. The therapists’ panel includes the following.

- Create and manage exercises through groups of gestures.
- Select single or multiple joints.
- Record a set of postures to be performed by the patient
- Select categories of groups to be performed for therapy.
- Select number of reps for each movement in a set or select a recursive time-based set.
- Assign therapies to existing patients.
- Monitor session-based patient performance.
- Suggest updated gestures based on current performance.

After the completion of each session, the system generates a report to the therapists which includes the percentages of the success rate, improvement, and fail attempts compared to other previous sessions.

**Figure 7:**
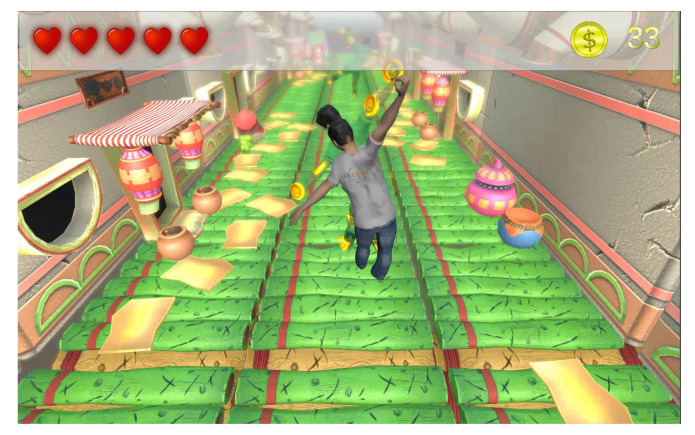
Screenshot of the gaming environment showing the patient character and the virtual assistant with different postures to be performed.

In order to access the patient’s dashboard, users have to log in first. The system has a profile for each patient, which saves all the prescriptions and exercises assigned to each individual by the therapist. Upon successful log in, the selected game is generated automatically with all the embedded exercises for that particular patient (see Figure 7). To play the game, a MS Kinect Xbox One is used to control the game via capturing the patient’s motions/gestures, together with an optional HTC VR headset which enhances the gaming experience. Using these devices eliminates the need for Joysticks and enables the penitents to move naturally while playing the games. At any moment, the patient is able to navigate to the dashboard in order to achieve the following.

- Receive notifications of newly assigned therapies
- Select and play several games after logging in
- Choose whether to use the VR headset (optional)
- Perform required exercises by matching postures from the virtual assistant, and by taking the multimodal instructions into consideration
- Notify the patient about the current status of successful or failed sets at a specific time or after each session
- Display incomplete therapy steps

It is worth noticing that the VR headset has been made discretionary to permit patients, who might feel discomforted when exposed to a completely virtual environment. A couple of side effects can be observed on a few patients, which are alluded to as “cyber sickness”, which includes dizziness, headache, disorientation, and postural instability affecting the patient’s overall performance [46]. Our system leverages the VR headset integration (HTC VIVE) with the Kinect in order to achieve a fully immersive experience. The position data and orientation tracking provided by the HTC Vive virtual reality device were combined with the body joint data captured by the MS Kinect, in order to generate a rich description of the human body motion. Nonetheless, the platform does not depend on the VR headset due to its complex control, particularly for patients who are diagnosed with disorder of upper limbs (i.e., cerebral paralysis or hand injuries). Thus, upper limb disorders involving the arms can be monitored, such as aches, pains, tension, and disorders involving any part of the arm from fingers to shoulders.

### 4.1. Intelligent Game Engine

When the game starts, various background processes/daemons start gathering Kinect frames on various body joints, direction data, and VR orientation. Data are continuously stored on the cloud to allow for expert monitoring, as well as real-time and sessions analytics. Different types of data are generated over time: (i) precalculated posture data that is generated based on the therapist inputs, including the defined parameters for each gesture and group of gestures, as well as the recorded postures for each therapeutic exercise, and (ii) the real-time patient data generated from both devices, which are saved with a high-frequency sensor producing up to 30 frames per second, following the specifications of the Kinect 2. Some of these frames can be dropped depending on the level of details required in the analysis.

RehaBot embeds a component for online game adaptations, which we call an intelligent game zone. These adaptation techniques are reflected in the algorithms described in Section 3. Adaptive configurations are progressively updated to the game profile in real-time based on patient’s performance. The new rules are then circulated to the configuration manager, which translates them into new adaptive postures that are performed by the virtual assistant. The injected virtual characters will display the required personalized posture that should be performed by the patient during the session. RehaBot takes into account the different vector dimensions and 3D angle rotations for all human body joints from both the ideal posture and the user current posture, in order to generate an adapted posture that matches and improves the user posture as much as possible. The virtual character can also adapt to users with special sitting positions or those using wheelchairs. Consequently, the game scene should be adapted with a matching character behavior and posture (i.e., sitting character). Therefore, RehaBot allows for capturing accurate posture data, and the results depend on the user context and experience.

**Figure 8:**
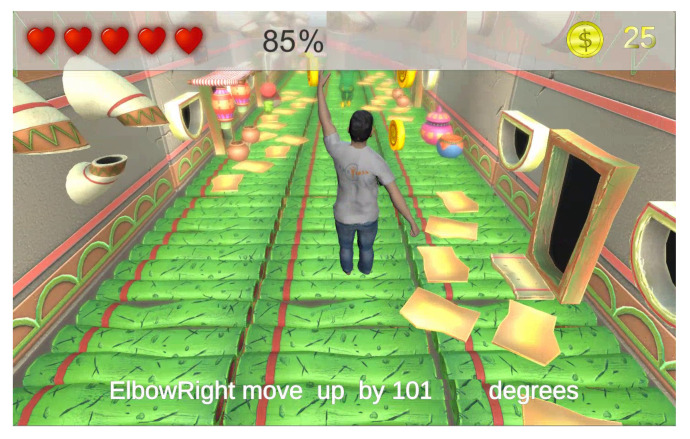
Alert is triggered with information on the gesture accuracy and instructions on how to improve.

Another sub-component of the game zone is the intelligent alerting responsible for generating instructions (i.e., voice and textual descriptions) for each matching process as illustrated in Figure 8. There is a high-level analysis (i.e., overall body posture) as well as a detailed performance assessment with the aim of guiding the patient to achieve a speedy recovery on each required exercise during the session.

One extra step beyond the posture matching and intelligent alert is the assessment of the overall exercise and individual gestures during one session, or multiple. The objective of the automatic correction component is to analyze each group of gestures and suggest adaptations to the difficulty level of that group with the aim of reaching the targeted performance. For example, a failure to achieve the target for a given exercise over several attempts will result in gradually decreasing the exercise difficulty, so that the target can be met by the patient in the next occurrences. Once the right posture is successfully recorded over three attempts, the component suggests a new set of exercises within the same group with a higher difficulty level until accomplishing the full set. A successful record on the full set allows for an adapted game strategy and scene with a more advanced level of exercises to be integrated, thus escalating the challenge to be met.

### 4.2. Advanced Analytics

Because RehaBot is designed as an adjunctive treatment that helps physiotherapist follow the patient progress in real-time and over sessions, instantaneous and session-based analytics are considered as a core component in the system. A deeper understanding and assessment of a patient’s performance can definitely help in drawing better conclusions and tailoring the game parameters to suit the patient’s behavior over sessions. Among many factors, the system monitors the response time in each exercise, body posture correctness and joints’ range of motion, thus maintaining an appropriate game difficulty level. Moreover, a modified or upgraded set of gestures will be proposed for the next sessions, while keeping a high standard of entertainment and personalization to achieve a rich and effective user experience. The mining techniques are used to search for consistent patterns as well as systematic relationships between the different input variables. For example, by knowing the user profile (e.g., age, gender, and preferences), and the physical challenges they are facing currently (e.g., upper/lower limb disorders, back pain, and hemiplegia), the therapist can define a few parameters on which our system can be based to enrich the game scenario with personalized configurations and adaptive exercises. The goal of the data mining is data prediction. For instance, we can use the mining techniques to predict improvement on a timeline by following a calculated schedule of exercises over sessions. Other measurements include (i) weekly- and monthly-based performance indicators; (ii) physical effort required and level of pain while performing the game-based exercise over a session, a week, or a month; (iii) effort vs. improvement analysis; (iv) VR vs. non-VR-based assessment; (v) reaction time and time to accomplish a given exercise; and (vi) number of practiced vs. cleared game levels, among others.

## 5. Implementation

**Figure 9:**
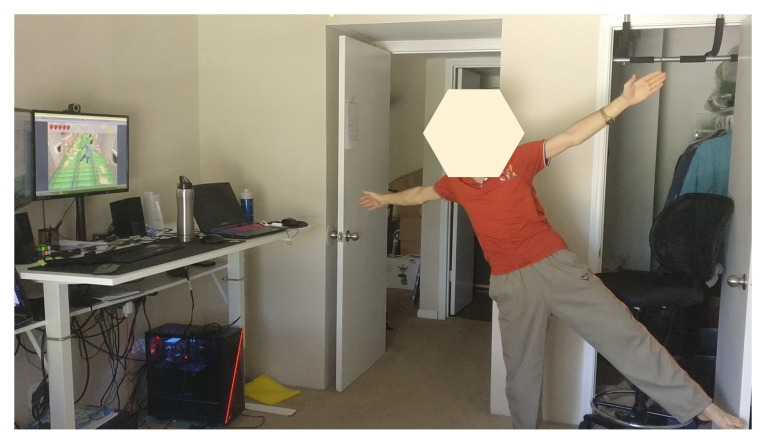
Screenshot of the setup environment showing one patient trying to replicate the virtual assistant posture.

We have developed an e-health platform that offers several advantages over traditional systems for therapy design and monitoring. Figure 9 demonstrates the setup environment with one user playing the game, by using the Kinect and VR devices. The user presented in this figure was a volunteer who does not specifically have a specific disability but, rather, deals with a mild lower back pain. Based on our discussion with experienced physiotherapists, we tested standard stretching postures and exercises that help in stretching and strengthening back muscles. As illustrated in the figure, the user is freely and naturally performing their required therapy without the need of any extra holders. Using the Kinect 3D motion capture sensor, the system is capable of tracking the movements of 25 joints in the body with up to 30 frames per second. This gives us the ability to record the motion of each joint over time as well as its Range Of Motion (ROM). Second, a Vive VR headset is coupled with the system to allow for a fully immersive environment, where users are engaged in a interactive 3D scene, and adaptive tasks are assigned to them according to their cumulative performance over time.

Within the platform, two applications have been developed to address the needs of therapists and patients. Both store their data and analysis on the same cloud environment. To use the application at home, patients need a computer linked to the internet, a Kinect device, and, only if desired, a VR headset. The patient logs in, selects the game, and starts playing. The therapist, on the other hand, accesses the authoring interface using a similar equipment to design, record, and assign personalized exercises to each patient. Particularly, the authoring interface enables the therapist to select the joints that need to be focused on using a model of a human body anatomy and then utilizes the Kinect sensor to record the appropriate postures. Both therapists and patients can replay any session in a 3D representation. The patient mainly uses this feature to check the ideal therapy postures recorded by the therapist, while the therapist can view the complete motions performed by the patients during the session. Moreover, both therapists and patients are able to visualize current or previous session’s performance improvement metrics plotted on a live graph.

Using the Unity 3D game engine, this adaptive game has been created. Although the algorithm has been developed to record data captured by the Kinect Xbox One and the HTC Vive headset, the game engine can be easily modified to support other 3D capturing devices of motion like the Intell Real Sense devices. The adaptation algorithm is continuously utilizing the JASON formatted precomputed data generated by the virtual assistant as well as the real-time data generated during the therapy session to create the next appropriate set of exercises/gestures based on the performance of the patient. The system gathers two types of real-time data: (1) the game data, and (2) the output of the 25 joints captured in each Kinect frame and the 3D angular rotation recorded by the Kinect devices. Kinect data consist of a sequence of frame IDs associated with time stamps. There are a number of properties for each frame, including the vector direction between any two adjacent joints (i.e., ShoulderRight⟶ElbowRight), the confidence level (a floating number between 0 and 1), the position of the joints, and the 3D angular rotation, among others. The game data, on the other hand, store computed positions of game’s objects, the collected coins, the repellent objects, and the virtual assistant’s positions and postures. It is worth noting that these positions are continuously adjusted in real-time depending on the capability of the patients together with the game’s level of difficulty. For instance, for a patient who uses a wheelchair, the height of the collected and repellent objects will be adapted to be attainable by that patient.

## 6. Evaluation

Different case studies can be considered to evaluate the performance of the proposed system, such as chronic neck pain, lower back pain, and scoliosis. With increasing medical costs, reduction in paid benefits, longer waiting periods, and a shortage of rehabilitation specialists, individuals with such diseases are in need of low-cost, quality, home-based sensorimotor rehabilitation. One of the main medical conditions which a lot of people encounter, and which requires a continuous monitoring in all periods and not only during therapy sessions, is non-specific chronic neck pain. Non-specific chronic neck pain is a disease of the neuromusculoskeletal system, which has postural or mechanical basis and affects between 30% to 50% of the general population in developed countries [47]. If it progresses, it can have serious consequences on the patient [48]. The following study will then focus on treatment of non-specific chronic neck pain due to its importance and the availability of patients. Our platform was also tested on a small number of lower back pain patients, and results were reported in our previous work [5].

### Methodology for the Clinical Study

After a discussion with specialized physiotherapists on the requirements for developing a comprehensive evaluation study to assess our platform, a series of exercises for stretching and strengthening neck muscles was proposed. Ten patients (6 male and 4 female) diagnosed with non-specific mechanical neck pain were recruited (i.e., neck pain continued for at least the last 12 weeks) to evaluate the intrinsic properties of the adaptive gaming platform, while four more patients were recruited to perform the traditional procedure with an active program for neck pain relief. The duration of the training program was two weeks (10 min, 10 sessions/2 weeks) for all patients. Recruited patients, with an age ranging between 18 and 50 years, were all office workers (with an average of at least six hours sitting in front of a computer screen), as studies have demonstrated that the highest annual prevalence of neck pain is shown among office workers [49,50]. To determine eligibility of participation, patients referred for inclusion were evaluated by a physiotherapist. Exclusion criteria include neck pain associated with vertigo, osteoporosis, vertebral fractures, psychological disorders, tumors, metabolic diseases, and previous neck surgeries. The evaluation process was conducted over two weeks with 10 sessions of 10 min duration.

**Figure 10:**
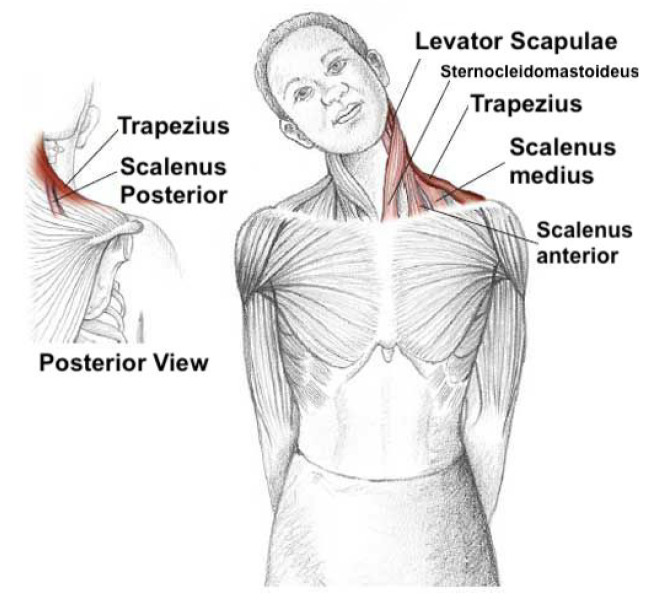
Lateral Neck Stretch: Main Neck and Shoulder Muscles.

**Figure 11:**
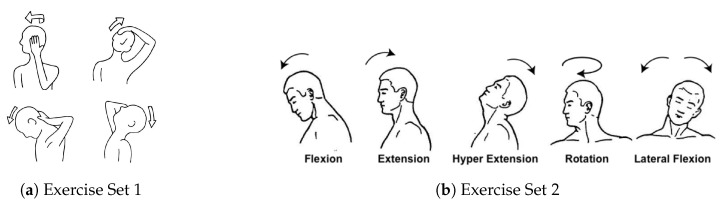
Two sets of exercises for stretching and strengthening of neck muscles.

Stretching and strengthening exercises include lateral flexion of the upper part of the trapezius, ipsilateral flexion and rotation for the scalene, and flexion for the extensor muscles, holding each movement for 10 s (see Figure 10 for neck muscles). Furthermore, Figure 11a shows the co-contraction flexors, rotators, and inclines, where a patient performs cranial nerve flexion, while the physiotherapist asks him/her to tilt, rotate, and look toward the same side while he/she opposes a resistance with his hand. There are several possible movements in the normal range of motion of the cervical spine (see Figure 11b). These are highlighted as follows.

- Flexion: a movement by which the chin attempts to touch the chest. The normal cervical range of motion for flexion is 80 to 90 degrees.
- Extension: starts with both shoulders relaxed and the head in a neutral position, then by moving the head to look up towards the ceiling (ROM up to 70 degrees).
- Lateral Flexion: happens when moving your head from one side to another, while trying to bend your neck to bring your ear down towards your shoulder. This movement is equal on both sides. The ROM is between 20 to 45 degrees on both sides.
- Rotation: starts from the center by looking forward, then rotates to look over you shoulder, then returns back to the center (works equally on both sides, with a ROM of 90 degrees).

Exercise sets 1 and 2 were assigned randomly to two groups of patients, and each group was evaluated based on the assigned set and by using the same evaluation criteria. Each exercise was automatically injected and repeated 5 times during a game session. Finally, a neck straightening exercise was performed by returning the head 5 times for 3–5 s. Patients were advised to perform the stretching program 5 times a week, with a single session taking about 8–10 min to perform.

## 7. Experimental Setup

The first phase is the experimental evaluation, which consisted in requesting the therapists to assess the effectiveness of the platform and to finalize the different metrics to monitor the patient performance in real-time and on session basis. Following on from the first phase, the platform was updated and made ready for a detailed evaluation of the platform.

The evaluation phase is designed to answer three main questions:

Q1. How efficient is the platform to perform its intended tasks?
Q2. How effective is the platform in fulfilling its intended use?
Q3. What is the user perceived value (satisfaction level) about the usability of the platform?

Both quantitative and qualitative methods are used to evaluate the effectiveness and usability of our system. In quantitative methods, different metrics were studied and monitored to analyze the quality of improvement of the patient, such as, the visual analogue scale (VAS) before and after exercising, and the range of motion, among others. The aim of these metrics is to assess the performance, reliability, correctness, and effectiveness of the system. Other metrics also include the following.

- Movement of the joints: it represents the therapy distribution impact over the body distinct joints. During the therapy session, the positions of each moving joint of the patient’s body are recorded. In fact, the Kinect device generates up to 30 frames per second, each frame contains the joint position and orientation in 3D axes. By taking the sequence of frames into account, one can identify the joint motion, orientation, and speed, thus providing accurate measurements regarding the range of motion and improvement of motor controls.
- Incorrect Body Gestures: One example of the incorrect body gestures is moving the forearm to practice a gesture that is supposed to be performed using the wrist. The system records the number of times an incorrect gesture is performed associated with a time stamp.
- Reaction Time: it is the time period between the instruction appearance and its implementation.
- Range of Motion (ROM): the system records the minimum, maximum, and average ROM of each therapy gesture along with the full session of the therapy. Measuring the ROM is done by recording the minimum and maximum position for a given joint and for each performed gesture. For example, if the monitored exercise is the neck lateral flexion (moving the neck to the right and left), the ROM will be determined from the recorded frames, starting from the initial neck position to the most flexed position as performed by the user. Then, the difference between the two vectors is calculated to determine the maximum ROM.
- Speed: It is defined as the velocity of the patient’s moving gestures during the game.
- Pattern Matching: it is defined as the accuracy of the matched patterns of each exercise and the full session.
- Success Rate: it is defined as ratio of the number of completed therapy sessions over the number of collected objects during the game.
- Error Rate: it is defined as the total number of therapy session that are not performed correctly over the number of total completed sessions.
- Hits: it is defined as the number of obstruction hits.
- Interest: it is defined as the engagement level in the game (how fast the patient gets bored of playing the game).
- Other Metrics: We are also calculating various other metrics that are needed by the therapist. These include the angular speed, angular rotation, and acceleration, which help in reporting the muscle power. The Visual Analog Scale (VAS) pain score is also determined before and after each session by filling a standard form by the patient for comparison purposes.

A qualitative evaluation has been conducted to measure the system’s usability and users’ satisfaction. Two separate sets of questions were prepared to be used appropriately with the therapists and patients. Each has questions about users’ perceived value of usability, adaptiveness, efficiency, enjoyability, fatigue, efficiency, level of motivation, and mental effort needed to use the system. The next section demonstrates the results of the qualitative questionnaires and feedback of the users.

## 8. Results and Discussion

Our objective lies in enabling patients to achieve and maintain the correct cervical alignment and sagittal balance. This requires performing stretching and balance activities for a sufficient period of time, while maintaining an accurate level of difficulty created and carefully adjusted for each exercise.

### 8.1. Correctness and Efficiency

System efficiency is evaluated using several metrics including joint-to-joint vector analysis for a given gesture, posture matching accuracy, gesture duration (i.e., reaction time and accomplishment of each gesture), exercise completeness, and percentage of hits for attractive and repellent objects (i.e., obstructions). Some of the metrics can be collected and analyzed from raw motion sensor data (e.g., posture matching), while other metrics are developed with samples of patient data over a session or multiple sessions, such as the mean time, duration, and joint range of motion evolution.

**Figure 12:**
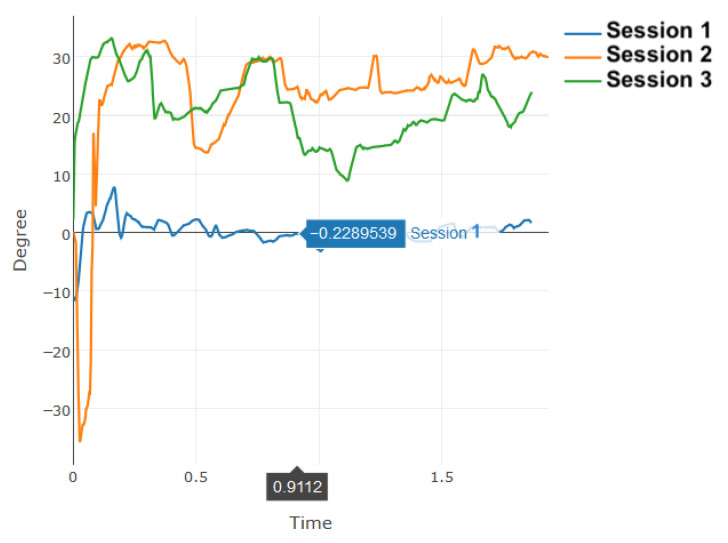
One patient’s Kinematic analysis over time of a lateral flexion exercise in different sessions.

**Figure 13:**
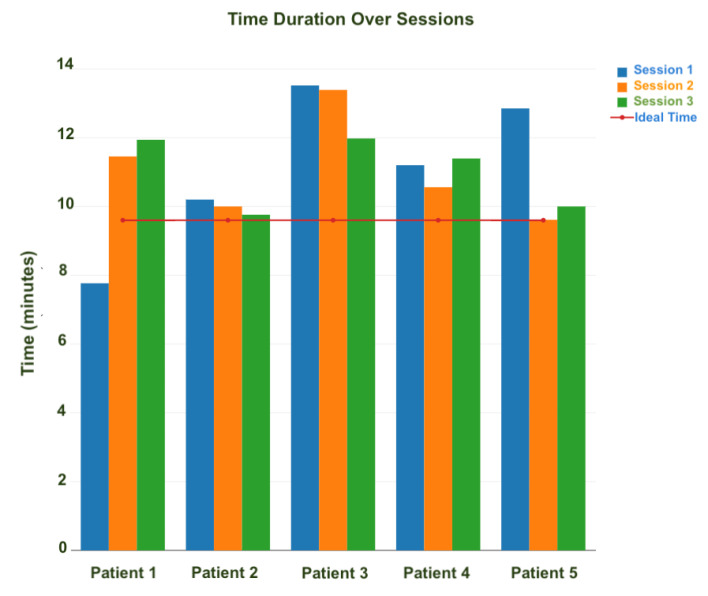
Total time required to finish the prescribed therapy in different sessions.

Figure 12 demonstrates a close monitoring of the range of motion for a lateral flexion gesture performed by one patient during a small time interval. This measurement was meant to show the accuracy of the calculated data based on the raw Kinect data. As illustrated in this figure, one can see the ROM ranges between 0 to 35 degrees in this case, which is normal as the maximum ROM possible for this action is 45 degrees. However, some records show negative results, which are noises produced in the collected Kinect frames. Most of these outliers are filtered out in the filtering process as discussed in Section 3.1. Figure 13 shows the total time for reaching a given set of exercises was calculated. For clarity purpose, only the results of five patients is shown in this figure, where patients were required to perform the same set of gestures with ideal completion time of 4 min. The common behavior seen here is that the learning curve is quick and patients were quickly improving their time records over sessions.

**Figure 14:**
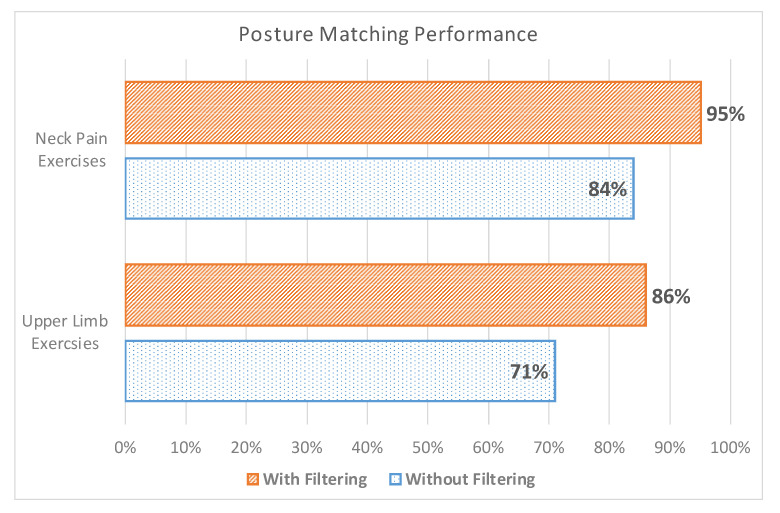
Posture matching accuracy performance before and after filtering.

Moreover, we designed an experiment to measure the accuracy of the posture matching algorithm by comparing its performance in detecting the right posture and detailed user gestures dynamically. For that purpose, we compared the original approach that uses the basic body tracking available in the Kinect 2 without filtering, against the approach that is based on the double-staged filter described in Algorithm 1. Figure 14 demonstrates our analysis based on two sets of exercises: (i) upper limb exercises and (ii) neck pain exercises. Upper limb exercises (e.g., for lower back pain, hand, and shoulder therapies) comprise more complex body gestures that can sometimes be challenging to detect due to overlapping joints from the Kinect perspective. The other factors that may affect the capturing performance are (1) the user going out of the field of view of the Kinect and (2) signal fluctuation. The results show a better performance by using the smoothing technique in both sets of exercises. The neck pain exercises are easier to detect due to no overlapping joints, and thus shows a high detection rate when using filtering because of the previously mentioned factors.

**Figure 15:**
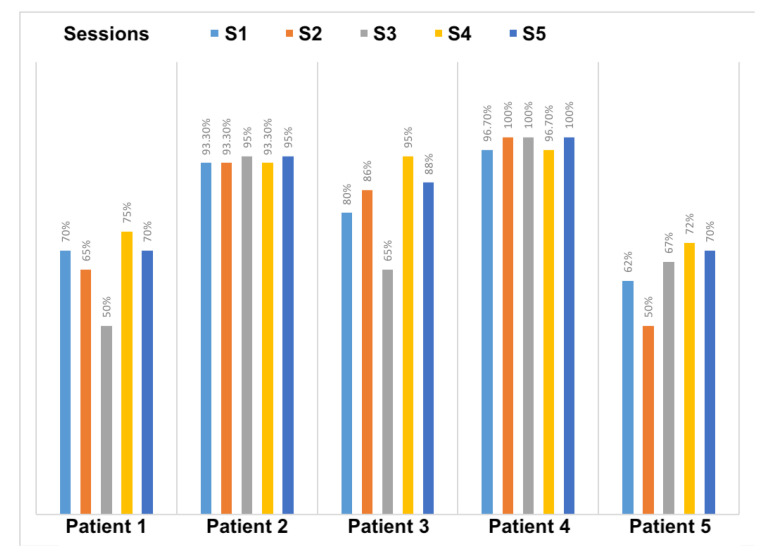
Percentage of achievement and correctness with respect the prescribed therapy.

On the other hand, we evaluated the average percentage of correct gestures performed by patients over different sessions. Figure 15 demonstrates the results of patient performance in each session. This result considers all the gestures required in a given prescribed therapy. The threshold adopted to consider a given gesture as correctly performed is 80%. Thus, gestures performed with a lower matching percentage are considered as being not acquired. The overall performance of the users shows a general increasing of correctness percentage, with some patients were performing better than others, mainly those who adhered to the gaming environment faster.

### 8.2. Measures of Effectiveness

For comparison with the traditional procedure, we selected four patients out of ten from those who are testing the gaming platform, and which have the closest conditions to the patients performing the traditional program. Three males and one female were assigned the exercise set number 2 in both groups (see Figure 11b). Both groups had an age range between 27 and 42, and did not present any chronic disease or other types of disability. Measurements were conducted in the same order: flexion, extension, hyperextension, rotation, and lateral flexion. All the tests were performed in the department of physical rehabilitation of the University of Sharjah by an experienced physiotherapist. To measure the outcomes of this program on both groups, the Visual Analog Scale (VAS) for pain level and the Neck Disability Index (NDI) were collected at three different period of time: (i) before starting the training program, (ii) after five session, and (iii) after ten sessions. The subjects involved in the study will record their pain intensity according to a VAS score. They should estimate their pain on a scale from 0 to 10, where 0 indicated “no pain” and 10 would be “the worst conceivable pain”. The NDI is an instrument that measures the functional status of subjects with neck pain within 10 categories, including pain, personal care, weight gain, reading, headache, concentration, work, driving, sleeping, and leisure. Each category is rated on a scale of 0 to 5, where 0 means “painless” and 5 means “the worst conceivable pain”. The resulting scores are added to a total average. The questionnaire is computed as an average percentage. The disability categories for NDI are 0–8%, without disability; 10–28%, mild; 30–48%, moderate; 50–64%, serious; and 70–100%, for complete disability [51].

**Figure 16:**
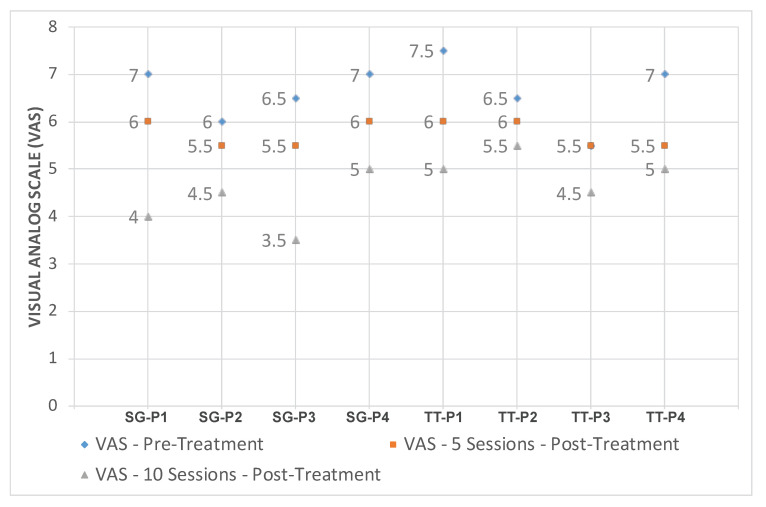
Evolution of Visual Analog Scale (VAS) score for patients using RehaBot vs. patients using traditional therapy.

**Figure 17:**
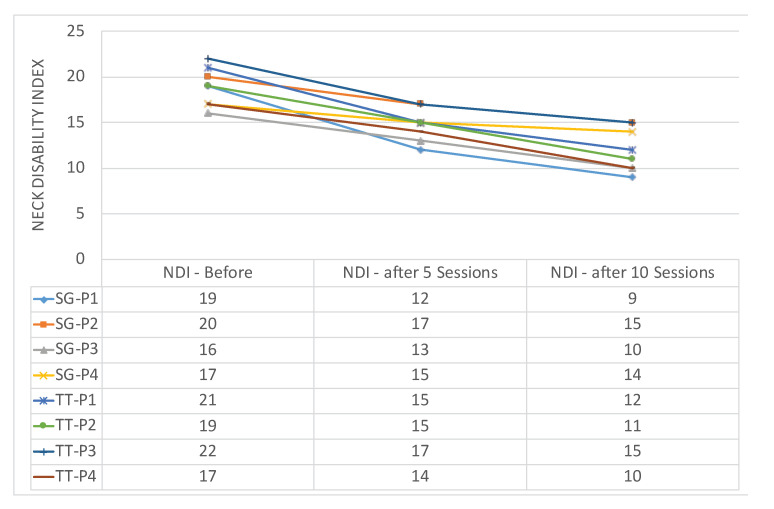
Neck Disability Index for patients using RehaBot (SG-P1 → SG-P4) vs. patients with traditional therapy (TT-P1 → TT-P4).

Figure 16 demonstrates the evolution of VAS score on patients of both groups, SG-P1 → SG-P4 for those who exercising using our serious games, and TT-P1 → TT-P4 for patients with traditional therapy. On the other hand, the NDI index for both groups is illustrated in Figure 17. The recruited subjects were having mild to moderate neck pain in the beginning, and they all showed a progress when exercising towards reducing their pain. We observed, however, that patients SG-P1 (male: 35) and SG-P3 (male: 27) were very engaged and performed their exercises more accurately, which resulted in having less pain and a decrease in their NDI index by the end of the ten sessions. It should be also noted that the group who performed the traditional program was closely monitored by an expert to ensure the correctness of gestures, which was not needed while using our platform.

**Figure 18:**
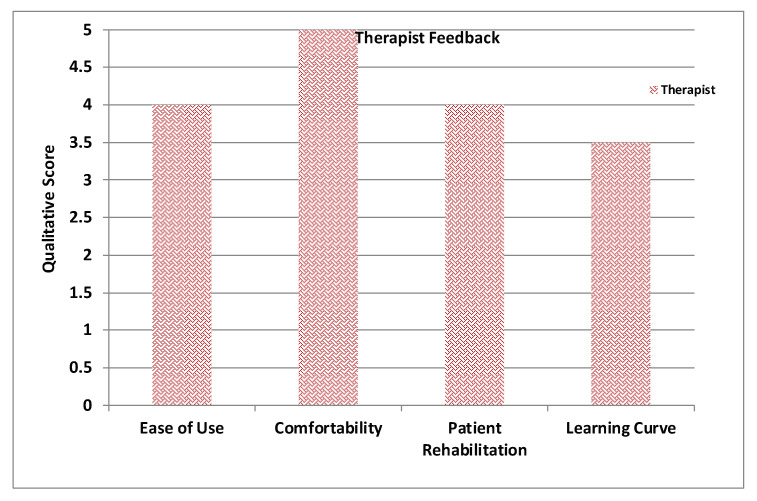
Average therapists’ feedback.

**Figure 19:**
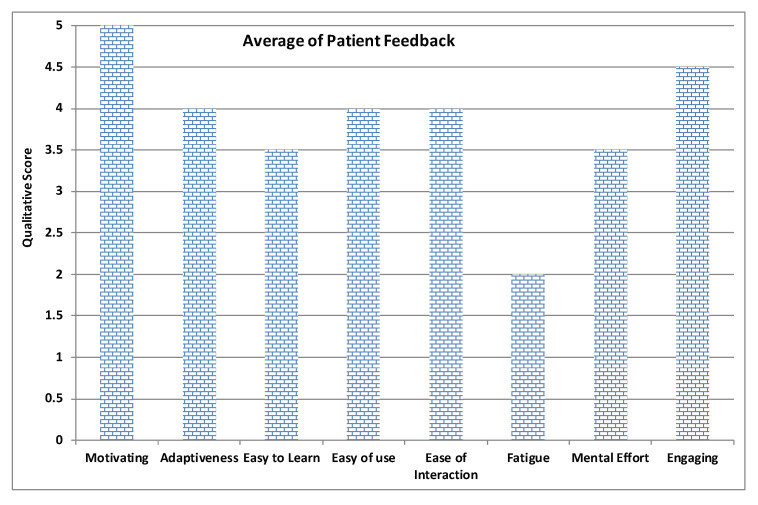
Average of patient feedback.

**Figure 20:**
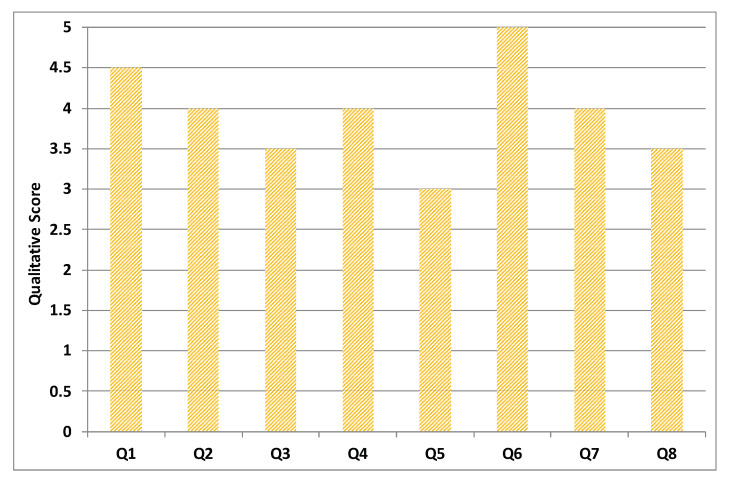
Average of patient feedback based on System Usability Scale Questionnaire (SUS) criteria.

**Figure 21:**
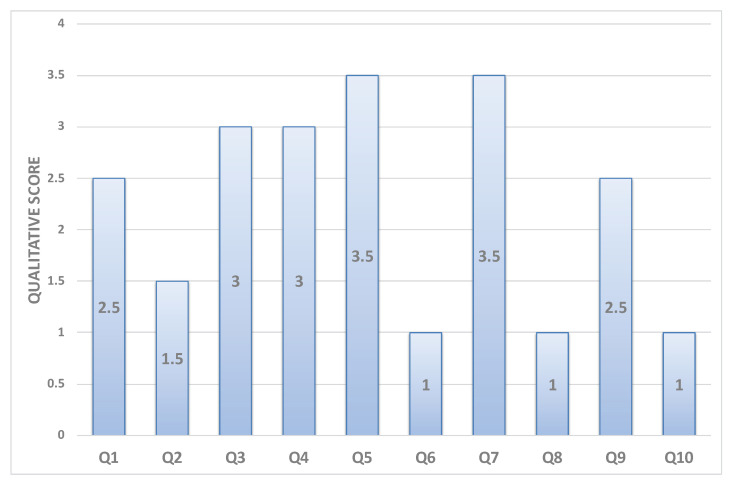
Average of patient feedback on adaptiveness.

In order to measure the usability and adaptiveness of the system, the ten patients who used our platform were also requested to fill out different questionnaires. Three types of self-administered, close-ended questionnaires were designed and distributed to the users. All questionnaires were written in English and it was considered that all users were capable of understanding the language. System Usability Scale Questionnaire (SUS) standard criteria were considered in the first questionnaire, which aims at assessing the learning curve and confortability while using RehaBot. Another specialized questionnaire was designed to measure the effectiveness of the adaptive platform on users’ performance. Results are highlighted in Figure 18, Figure 19, Figure 20 and Figure 21. Other questionnaires are going to be considered in the full clinical test to evaluate the gamefulness of our system such as the one proposed in [52].

Figure 18 illustrates the average feedback given by the therapists who devised the set of gestures and tested the features in the therapist dashboard including creating exercises with several groups of gestures, assigning therapies to patient, and monitoring patient performance. Figure 19 displays the average of patient feedback with respect to several usability measures such as enjoyability, learnability, ease of interaction, etc. Figure 20 illustrates the average patient feedback on the System Usability Scale Questionnaire (SUS) as a standard and reliable tool for measuring the usability of our platform. The questions of the SUS questionnaire are presented in Annex A. The questions are a mix between positive and negative questions, and the scale is between 0 for strongly disagree, and 4 for strongly agree.

Finally, Figure 21 demonstrates how patients have perceived the adaptiveness of our system with respect to several factors. The questions Q1–Q8 that are shown on the X-axis represent the eight-questions of the questionnaire as follows. Q1. *The virtual assistant guides me to do the right posture*. Q2. *The automatically generated set of exercises exactly reflect my needs*. Q3. *The system understands and adapts to my capabilities and performance*. Q4. *The multimodal instructions help me to understand my mistakes*. Q5. *The instructions provide me with clear suggestions on how to improve my gestures*. Q6. *The system helps me to be more effective*. Q7. *I can recover from mistakes quickly and easily*. Q8. *The adaptive virtual assistant adapts the posture difficulty, and this makes me feel more confident*.

All plots demonstrate satisfaction results on a scale from 0 to 5. In general, most metrics show satisfactory results, although the learning curve was mentioned as an important factor that could affect the initial sessions performance, and this can be improved by increasing the number of training sessions for both therapists and patients.

### 8.3. Discussion

Both types of users demonstrated a high level of confidence in utilizing RehaBot and believed it can play a significant role in remote rehabilitative therapies. The motivation and engagement of participants while using our serious games was significant, which allowed for longer periods of practice. As a result, improvements in alignment has been associated with improved outcome of the treated neck muscles. We also took into consideration the recommendations suggested by the therapists and participants with respect to the user interface and the admin dashboard, as well as the “how” alerts and instructions displayed on the patient’s dashboard.

Furthermore, when comparing the results of efficiency and correctness between traditional therapy and using our system, both groups showed close performance. However, all patients agreed that performing such exercises at home using our platform would be much more convenient, as it is more engaging and ensures the correctness of performed gestures. Therefore, our findings support the feasibility and impact of using an adjunctive treatment with online adaptive rehabilitation, thus augmenting usual care for persons with chronic neck pain in a clinical context with potential similar role in continuity of other upper limb rehabilitation.

#### Limitations

This study investigated the feasibility and effectiveness of adaptive serious games developed for body rehabilitation for patients with musculoskeletal disorders. Although the results presented are promising and provide initial evidence on the impact of the approach in online rehabilitation, the experiments were limited to rather a small sample size and only on patients with chronic neck pain disease. Therefore, effectiveness and validity of the approach will have to be further tested on a larger-scale randomized controlled trial, and on other upper limb disorders, as well as for lower limb rehabilitation.

## 9. Conclusions

We present an adaptive serious game platform for patients with musculoskeletal disorders, referred to as “RehaBot”. This platform allows experts to remotely prescribe personalized therapies by only recording a screenshot of posture required and the exercise duration. RehaBot digitizes those postures and creates virtual characters, called rehab bots, that are injected within the serious game environment. These bots dynamically act as an adaptive coach and adapt themselves gradually according to patient’s performance. RehaBot presents an intelligent alerting and auto-correction system that adapts the level of game difficulty while controlling game configurations and objects in real-time. RehaBot embeds advanced AI and pattern matching techniques to build adaptive and personalized serious games, also referred as exergames. These exergames not only allow for an entertaining experience, but also pave the way for an effective training and rehabilitation. The prototype has been developed, and a clinical study was conducted with a number of patients and under the supervision of three physiotherapy consultants. To the best of our knowledge, RehaBot is the first fully-fledged, low-cost online platform for helping patients with physical disabilities, while naturally embedding fully adaptive assistant within the gaming framework for tracking and guiding patients’ progress over therapy sessions. This platform can also be applied to fitness training domain, where athletes can perform personalized and adaptive training with the help of the embedded virtual coach.
